# Stellate Nonhereditary Idiopathic Foveomacular Retinoschisis: Cataract Surgery

**DOI:** 10.1155/2022/7404138

**Published:** 2022-09-09

**Authors:** Sofie Van der Auwera, Oscar Kallay

**Affiliations:** ^1^Department of Ophthalmology, Antwerp University Hospital, Edegem, Belgium; ^2^Centre Médical de l'Alliance, Braine l'Alleud, Belgium

## Abstract

**Purpose:**

We present the first case described in the literature of cataract surgery in a patient with stellate nonhereditary idiopathic foveomacular retinoschisis (SNIF).

**Methods:**

In this case report, we describe the extensive workup we did on our patient, including optical coherence tomography, fundus autofluorescence, fluo-angiography, full field electroretinogram, and genetic testing. We moreover describe the cataract surgery with clear lens extraction and implanting of a multifocal implant.

**Results:**

The refractive and lifestyle profile of our patient made implantation of multifocal intraocular implants the only solution for this case. During preoperative measurements, a SNIF diagnosis was suspected after optic coherence tomography imaging which led to an even more extensive workup of our patient and the diagnosis of stellate nonhereditary idiopathic foveomacular retinoschisis. We then proceeded to cataract surgery, which was performed safely and without any sequellae.

**Conclusion:**

Stellate nonhereditary idiopathic foveomacular retinoschisis (SNIF) is a relatively new disease entity. Only few cases in the literature describe this disease, and none of them describe cataract surgery in a patient with SNIF. We therefore describe the first known cataract surgery in such a case. Long term follow-up results show that the procedure can be performed safely. *Summary Statement*. We hereby present the first case described in the literature of cataract surgery of a patient diagnosed with stellate nonhereditary idiopathic foveomacular retinoschisis. A clear lens extraction with implantation of a multifocal intraocular implant was conducted with excellent postoperative results and a happy patient.

## 1. Introduction

Stellate nonhereditary idiopathic foveomacular retinoschisis is a rather new diagnosis in the retinal ophthalmology field. A few case reports about the disease entity have been published. Since little is known about SNIF, there is currently no data present about the postoperative status of anterior segment surgery with phacoemulsification in these patients. We hereby present a 51-year old woman with stellate nonhereditary idiopathic foveomacular retinoschisis who underwent a clear lens extraction for refractive needs and her 1-year follow-up.

## 2. Case Report

A 51-year old woman consulted for advice concerning refractive surgery. She had an intolerance to progressive glasses and suffered from daily work as a hairdresser. She was currently wearing monofocal glasses with a spherical correction of +3.50 on both sides, which resulted in a Snellen visual acuity (VA) of 20/30, without possible improvement and poor near vision. The autorefractor values were OD +4.00 (-1.25@97) and OS +3.75 (-0.50@90). Best corrected VA for near was Parinaud (P) 3 for both eyes. Clinical examination nor biomicroscopy did reveal any particulars. Fundoscopy was normal.

**Figure 1:**
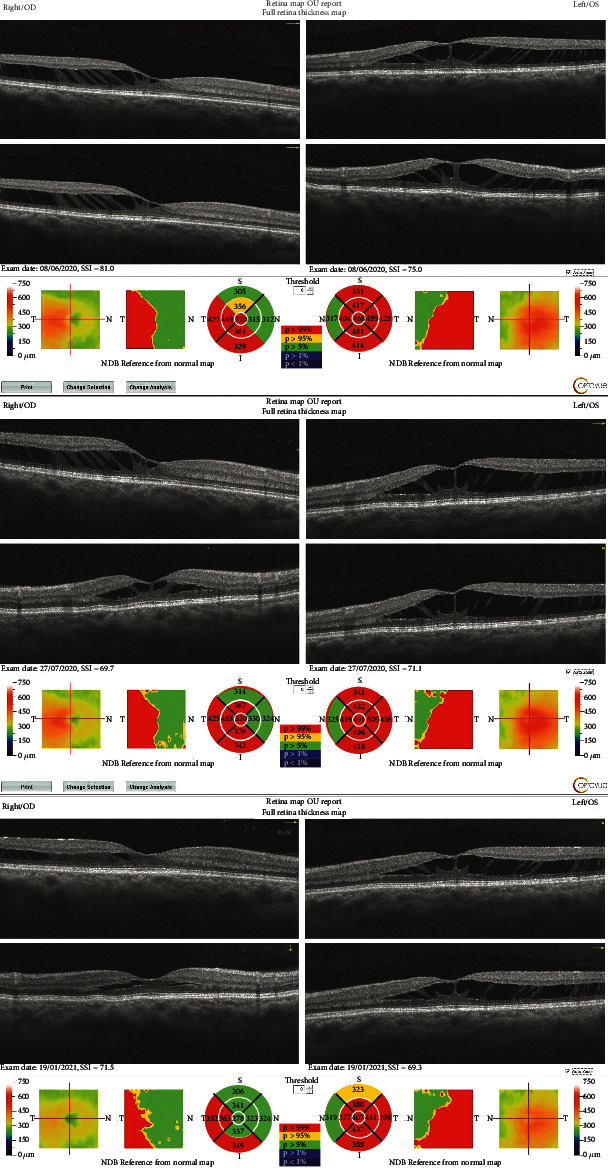
Bilateral OCT-imaging preoperative, 1 day postoperative and 6 months postoperative.

A macular optic coherence tomography (OCT) was performed which showed a bilateral stellate retinoschisis pattern between the outer plexiform layer and the outer nuclear layer. The schisis was located macular and was more widespread in the left eye.  Figure 1.

**Figure 2:**
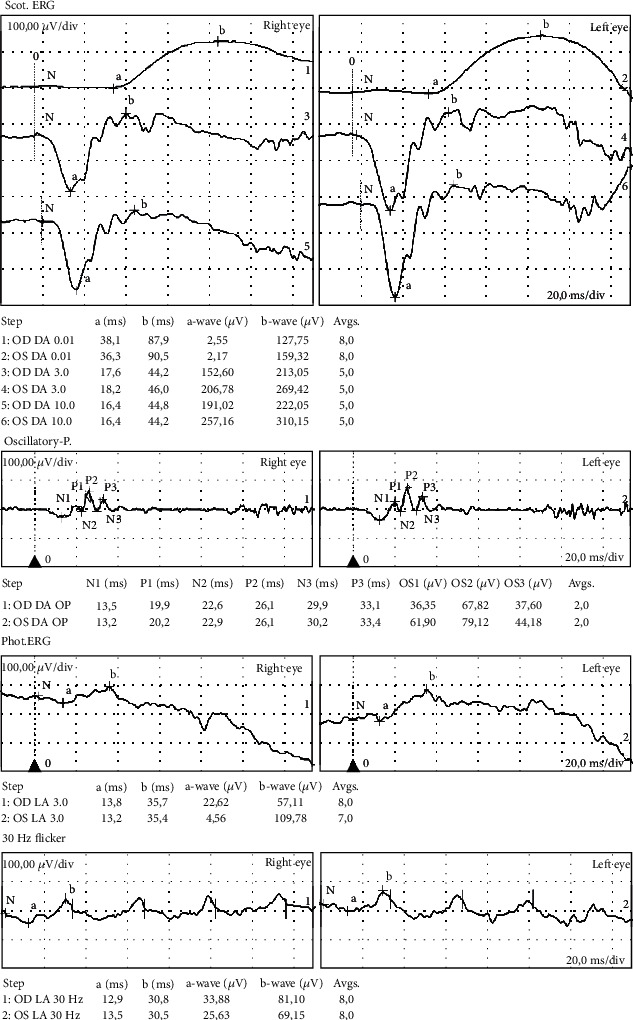
Full field ERG results.

Some additional tests were done to determine the origin of the retinoschisis. These tests included fundus imaging (Clarus®), fluo-angiography, and fundus autofluorescence. Each of these examinations showed normal macular anatomy and normal retinal vasculature without obstructions or hemorrhages. No exudates were visible. The optic disc had an estimated cup-disk ratio of 0,5 OD and 0,3 OS. A full-field electroretinogram (ff-ERG) was performed and showed some lowering in the amplitudes of the scotopic ERG, especially on the b-wave, but no electronegativity was seen. The photopic ERG showed higher amplitudes for the cones. These ERG-results correspond with the schisis seen on OCT, leaving the photoreceptors in contact with the outer nuclear layer (Figure 2). Blood samples for genetic testing were obtained, which showed our patient to be RS-1 negative. Thus excluding the possibility this retinoschisis was part of a known X-linked retinoschisis syndrome [1]. OCT-imaging in our patient parents showed a normal bilateral retina without schisis or other disease indications.

These additional results pointed towards the diagnosis of stellate nonhereditary idiopathic foveomacular retinoschisis (SNIF). SNIF is more often seen in female patients without hereditary mechanisms to explain the retinal disease. Typically, there is a rather low decrease in vision that does not cause any complaints. The schisis is, as suggested by its name, foveomacular without peripheral involvement. All of this pointed us in the direction of SNIF.

Being a hairdresser, a good vision for both near and far was necessary for the patient, but correction with multifocal glasses nor with contact lenses was tolerated. Because the refractive needs of our patient were not solvable with noninvasive refractive methods, we decided to take a leap and operate on our patient with SNIF.

When looking into literature, such an operation has not been described and thus was performed without knowing how the eye would react.

Phacoemulsification with a placement of multifocal implants was performed to meet the refractive demands of the patient. A superior scleral incision was used after which a manual capsulorhexis was performed. Phacoemulsification with the signature phaco machine was performed while using a linear flow. After removal of the clear lens and cleaning of the lens capsule, an implant was placed intracapsular. We used the AT LISA tri 839MP implant from Zeiss with bilateral +25.00D power. Peroperative lidocaine hydrochloride 1% and cefuroxime 10 mg/ml were used. A postoperative treatment regimen with dexamethasone eye drops, tobramycine eye drops, and indomethacin eye drops was prescribed.

The postoperative status led to a monocular vision of 1.2 (20/16) OD and 1.0 (20/20) OS, an improvement of, respectively, 2 and 3 lines on a standard Snellen vision chart, and a monocular near vision of P2 on both sides. Standard postoperative treatment with dexamethasone, tobramycine, and indometacine was augmented with nepafanac 3x/d to protect the retina from further damage. When consulting again after 6 months for a postoperative control, the vision had once again decreased to a best corrected Snellen VA of 0.7-2 (20/30) OD and 0.6 (20/30) OS and near vision P3 OD and P4 OS. At the 1-year postoperative control, the vision remained stable compared to the last control with no subjective complaints of decreased vision. Between both controls, autorefractor values remained stable as well with OD +0.75 (-0.75@94) and OS -0.25 (-0.25@150). The OCT images remained also stable (Figure 1). The patient was very happy in her daily work and private life with her eyesight.

## 3. Discussion

A stellate macular retinoschisis pattern in an RS1-negative patient in an eye without any other anatomical or functional defects is rather rare and can be classified as SNIF. Typically, this disease category represents stellate retinoschisis patterns in the foveomacular regions in an RS1-negative patient without a known family history of retinal diseases [2–5]. SNIF is a rather new diagnostic entity, with few case reports. Shimazaki and Matsuhashi [6] described a similar clinical entity as well. Most interestingly, the ERG-findings seem to be very similar between our case and theirs, with normal findings, but however a sensitive decrease of the b-wave. Their case describes a mother and daughter with the same type of retinoschisis in a SNIF pattern. In our case, however, additional OCT imaging of both parents was negative. Moreover, retinal abnormalities could be seen on fundoscopy in both women, which was not the case with our patient.

None of the described cases in literature underwent cataract surgery at the time. It is the first report of a phacoemulsification in a patient with SNIF. We had good postoperative results, without worsening of the schisis pattern. However, 1 patient is too few to draw any conclusions. We hope that when other cases in the literature age and their refractive need change, the results of the phacoemulsification procedures will be bundled and published.

Moraes et al. [7] did however perform a vitrectomy with phacoemulsification in a patient with SNIF and published about this some months after we treated our patient. They also concluded this procedure could be performed safely. They present postoperative results quite similar to ours, with no subjective decrease in vision and a good overall outcome. They did however not use a multifocal intraocular lens but a monofocal one.

We do agree with Ober et al. and Shimazaki and Matsuhashi that the disease entity described is a new clinical entity within the larger group of retinoschisis [2, 6]. We agree that further genetic testing of the uncoded parts of the RS-1 gene could help understand the etiology of such disease. Extensive screening of other family members could also be helpful, as well as the screening of (male) children of these patients. We think the ERG is an important test in the diagnostics of this type of retinoschisis, however, its exact interpretation is not fully understood [8]. We hope that with further evolving technologies in the field of electrophysiology.

## 4. Conclusion

We present a rather rare case of SNIF in a 51-year old female patient with bilateral disease in hyperopic eyes. Cataract surgery with clear lens extraction was performed, resulting in satisfactory postoperative results without worsening of the disease status. It is our opinion that operating on patients with SNIF can be performed safely, if all necessary precautionary measures are taken.

## Data Availability

Comments and questions about the case can be sent to oscar_kallay@yahoo.fr.

## Additional Points

*Summary Statement*. We hereby present the first case described in the literature of cataract surgery of a patient diagnosed with stellate nonhereditary idiopathic foveomacular retinoschisis. A clear lens extraction with implantation of a multifocal intraocular implant was conducted with excellent postoperative results and a happy patient.
